# Sense and sensibility: of synthetic biology and the redesign of bioreporter circuits

**DOI:** 10.1111/1751-7915.13955

**Published:** 2021-10-24

**Authors:** Shimshon Belkin, Baojun Wang

**Affiliations:** ^1^ Institute of Life Sciences the Hebrew University of Jerusalem Jerusalem 9190401 Israel; ^2^ School of Biological Sciences University of Edinburgh Edinburgh EH9 3FF UK; ^3^ Hangzhou Innovation Center and College of Chemical and Biological Engineering Zhejiang University Hangzhou 311200 China

It is tempting to speculate that sixty years ago, when Jacob and Monod presented their model of the *lac* operon (Jacob and Monod, 1961), they already had a glimpse of the future of the *lacZ* gene, not only as encoding a cleaver of disaccharides, nor as a component in a beautiful and groundbreaking model of gene regulation, but also as a universal reporter of gene activation. Indeed, reporter gene technology rapidly became a basic tool in studying the regulation of gene expression; several decades had to pass, however, before the same approach has led to the first report of a microorganism genetically engineered to perform an accurate, specific and sensitive analysis of an environmental pollutant (King *et al*., 1990). The term ‘whole cell biosensor’ soon entered into use, accompanied by some semantic controversy: purists view the term ‘biosensor’ as a hardware device, in which the biological entity (e.g. enzyme, antibody, oligonucleotide or a live cell) serves as its sensing component (IUPAC, 2017); according to this view, a microbial strain, notwithstanding the complexity of its re‐engineering, may be called a ‘sensor strain’ or a ‘bioreporter’, but never a ‘biosensor’. Long before this linguistic polemic became an issue, however, a pioneering article from the Sayler group (King *et al*., 1990) described a bioluminescent *Pseudomonas*‐based sensor of naphthalene. This publication was trailed by the first *E. coli*‐based mercury sensor (Selifonova *et al.*, 1993), soon to be followed by numerous others, all sharing the same basic structure: a gene promoter induced by the target compound (directly, or via the removal of a repressor), fused downstream of a reporter gene. The latter could code for a traceable protein (e.g. GFP) or – more often – for an enzyme, the activity of which could be monitored quantitatively in real time (van der Meer and Belkin, 2010). When necessary, regulatory elements had to be cloned as well, especially when the gene promoter acting as the sensing element was not native to the host organism. In view of the practically infinite number of gene promoters and regulatory proteins available as candidate sensor elements, the scope of possible sensing targets of such sensors is exceptionally broad. In parallel to the development of microbial sensors of specific compounds, bioreporter strains have also been described for the detection of global sample characteristics such as toxicity or genotoxicity/mutagenicity, parameters of importance for environmental health as well as for chemicals’ safety. The commercial SOS Chromotest (Quillardet *et al*., 1982), the forerunner of this group of assays, was followed by the *umu*‐test (Oda *et al*., 1985). In both cases, the activation of gene promoters from the *E. coli* SOS repair regulon by DNA damaging agents was chromogenically monitored with *lacZ* as a reporter gene.

Looking back over the last 15 years, possibly the most powerful innovator of microbial biosensor design was the coming of age of synthetic biology. While the term has been introduced to the scientific literature over a century ago (Leduc, 1910), its meaning has slowly changed over the years. Following the introduction of the Jacob and Monod model, microbial biotechnology horizons opened up with the advent of increasingly more sophisticated molecular tools, including numerous enzymes derived from diverse microorganisms and viruses, harnessed and retrained to perform cutting, pasting and editing tricks. The same horizons practically exploded when thermophilic variants of these enzymes were ingeniously employed in the invention of PCR technology, and turned essentially limitless when genome sequencing was made trivial and bioinformatic data (and tools for its analysis) became freely available to all. These advances have prepared the ground for the invasion of practitioners of additional disciplines into the realm of whole cell sensor design; when engineers, physicists and computer scientists started to practice biology in earnest, things have started to become truly interesting. In 2004, van der Meer *et al*. have claimed that one of the reasons current bioreporters’ performance cannot comply with environmental detection standards is the ‘lack of engineering principles’. More or less at the same time, the ‘Biobricks’ concept has been presented (Knight, 2003), aiming to provide ‘a set of standard and reliable engineering mechanisms to remove much of the tedium and surprise during assembly of genetic components into larger systems’. The trend embodied by these two examples paved the ground for engineering school graduates to advise ‘classical’ molecular biologists involved in microbial bioreporter design that the time of simplistic promoter‐reporter fusions is over; more complex (and hopefully, more efficient and diverse) molecular sensor circuits can be designed, for both *in vivo* and *in vitro* expression, by employing an engineering‐like point of view.

Indeed, synthetic biology adopts engineering principles (e.g. standardization, modularization and modelling) to facilitate complex genetic circuit construction, particularly using ‘Lego‐like’ standardized building blocks (Endy, 2005). Though the blocks alone do not perform advanced actions, they can generate bespoke coordinated functions when connected. Hence, synthetic biology offers new tools to precisely manipulate cells for achieving customized tasks, using engineered gene circuits of varying scales and complexity. The developments in synthetic biology have permitted both fine‐tuning the performance of existing microbial biosensors, and creating new ones with unique functionalities in a more predictable and rapid manner.

Synthetic microbial biosensors typically comprise three exchangeable modules: an input sensing block, an internal signal processing block, and an output reporting block (Wang and Barahona, 2013). In contrast to traditional microbial sensors consisting of a genetic reporter fused to an inducible promoter to control the expression of a detectable output, synthetic biology enables biosensor designs to incorporate additional complex signal processing circuits. Accordingly, the sensing unit triggers more sophisticated actions before activating reporter expression, in order to enhance a sensor’s performance or perform additional functions. Such circuits include toggle switches (Gardner and Cantor, 2000), logic gates (Anderson *et al*., 2006; Wang *et al*., 2011), transcriptional amplifiers (Wang and Barahona, 2014) and memory circuits (Courbet *et al*., 2015; Riglar *et al*., 2017). Furthermore, microbial sensor cell arrays could be designed to display an easy‐to‐interpret output pattern corresponding to specific input analyte levels without the use of specialist lab equipment (Wan *et al*., 2019).

As many early stage microbial biosensors are inadequate to meet practical requirements in detection limit, specificity and output amplitude, various gene circuit‐based optimization strategies have recently been developed to improve their sensing performance. In contrast to traditional optimization methods such as random mutagenesis, these synthetic biology‐enabled optimization tools are based on rational design, and are thus more predictable and faster to achieve the desired sensing specifications. For example, integrating multiple signal inputs using genetic AND gates have been shown to be effective in increasing microbial sensors’ specificity (Wang *et al*., 2013), and rationally tuning the intracellular levels of the receptor proteins can drastically improve sensors’ detection limits (Wang and Barahona, 2015). In addition, a toggle switch (Wu *et al*., 2009) and a post‐translational regulation device (Wan *et al*., 2019) have been designed to lower microbial sensors’ background expression and detection limits. Amplification of the transduced sensor signal is another powerful strategy to further boost the sensor’s performance, using strategies such as positive feedback loops (Jia *et al*., 2019) or transcription signal amplifiers (Wan *et al*., 2019).

Albeit successful proof‐of‐concept laboratory demonstrations of a number of synthetic microbial sensors, very few have made it into the market. Several barriers remain to be overcome, including an insufficient number of sensory building blocks, poor sensing performance, long‐term stability issues, risk of releasing genetically modified microorganisms (GMMs), and lack of practical experience in acceptance by professional stakeholders (Hicks and Bachmann, 2020). Nevertheless, synthetic biology has contributed novel strategies to address these limitations. For example, different approaches have been applied to keep biosensor cells alive and active for longer term including freeze‐drying of cells, and encapsulating cells within polymers (Bjerketorp *et al*., 2006; Liu *et al*., 2018; Wan *et al*., 2019; Shemer *et al*., 2020). Recent advances demonstrated the potential of harnessing the amazing sensing capabilities of microbes for versatile applications, for example as wearable sensors for biomarker analysis in sweat to achieve non‐invasive in situ real‐time physiological state monitoring (Liu *et al*., 2018; Nguyen *et al*., 2021), or the standoff detection of buried landmines (Belkin *et al*., 2017). However, biosafety concerns regarding the usage of GMMs remain an issue associated with field and in vivo applications, including potential horizontal gene transfer and disruption of natural ecosystems. Accordingly, different biocontainment strategies have been proposed to mitigate biosafety concerns such as replacing antibiotics resistance with toxin‐antitoxin systems (Wright *et al*., 2015), incorporating conditional kill switches (Chan *et al*., 2016) and non‐canonical amino acid substitution (Fredens *et al*., 2019). Furthermore, chromosome‐free bacterial chassis such as SimCells (Fan *et al*., 2020) can be considered. Notably, cell‐free expression systems have become increasingly popular as a new sensor platform, by avoiding biosafety concerns associated with using living cells. Cell‐free biosensors lend faster responses, higher sensitivity and an enhanced compatibility to toxic samples (Lopreside *et al*., 2019; Silverman and Karim, 2020). Moreover, cell‐free extracts comprising genetic sensors could be embedded on paper, providing a portable platform for easy‐to‐use and cost‐effective on‐site screening (Pardee *et al*., 2016) or in hydrogels acting as smart stimuli‐responsive biomaterials (Whitfield *et al*., 2020).

The latest developments in synthetic biology enable a fast design‐build‐test cycle for sensor construction and response optimization, to address the limitations of microbial biosensors. Yet, challenges remain to be addressed both within and beyond the scope of technical developments. For instance, environmental, food and health monitoring necessitate sensor cell exposure to complex samples, and therefore require complex signal processing circuits and likely multiple input modules. Notably, for biomedical applications involving complex media compositions such as tumours, non‐specific localization of sensor cells prevents accurate diagnosis and biotherapy. Consequently, engineering microbes for sensing and reporting at designated spatial locations will be critical (Chien *et al*., 2021). Considering that a single microbial cell has a limited capacity in resources, and that large complex circuits tend to burden host cells, cell consortia comprising multiple communicating sensor strains may be used to facilitate multiplex detection and reconfigurability of sensor function (Wang *et al*., 2013; Khatun *et al*., 2018). Innovative designs with fewer time‐consuming signal propagation steps such as a transcription‐only design, with RNA as the reporter entity, or engineered ligand‐responsive fluorescent reporter proteins, could significantly shorten the response time of microbial biosensors, while alternative reporting formats such as direct bioelectronic signal output may lead to increasing seamless interfacing with conventional electronic devices. Further, integrating engineered microbial biosensors into various materials will lead to progarmmable living materials with bespoke functionalities such as self‐healing.

In summary, we have witnessed a new wave of microbial sensors development in the rising era of synthetic biology, and expect this trend to continue and probably grow stronger in the coming decades. While living bioreporters presently face certain restrictions, synthetic biology offers new tools and strategies to accelerate the development, enhance the performance and address the current limitations of microbial biosensors; this will facilitate their future adoption and uptake as promising alternative analytical devices in diverse settings.

## Funding Information

B.W. acknowledges support by the UK Research and Innovation Future Leaders Fellowship [MR/S018875/1], Leverhulme Trust research project grant [RPG‐2020‐241] and US Office of Naval Research Global grant [N62909‐20‐1‐2036]. S.B. was partially supported by the Minerva Center for Bio‐Hybrid Complex Systems.

## Conflict of interest

None declared.
